# Unique Case of a Refractory Esophageal Peptic Stricture in an Uncontrolled Diabetic

**DOI:** 10.7759/cureus.30236

**Published:** 2022-10-12

**Authors:** Vincent Wong, Anjella Manoharan, Dayna Panchal, Weizheng Wang

**Affiliations:** 1 Internal Medicine-Pediatrics, Rutgers University New Jersey Medical School, Newark, USA; 2 Gastroenterology and Hepatology, Rutgers University New Jersey Medical School, Newark, USA

**Keywords:** esophageal stricture, gastroparesis, diabetes mellitis, gastroesophageal reflux, esophageal stenosis, peptic esophagitis

## Abstract

Esophageal strictures can lead to the narrowing of the esophagus and dysphagia. They are termed peptic strictures when caused by acid reflux and usually measure less than two centimeters in the lower esophagus. Peptic strictures can be treated with proton pump inhibitors, endoscopic dilation, and esophagectomy. We present a unique case of a young diabetic who developed progressive dysphagia and was found to have a 5-centimeter esophageal peptic stricture refractory to treatment. His symptoms were secondary to gastroparesis and acid reflux from uncontrolled diabetes. Since diabetics are more likely to develop such complications, an important part of the management of peptic strictures should be focused on diabetes control.

## Introduction

Esophageal strictures are common structural lesions that are often amenable to therapy [1]. They can be caused by acid reflux, caustic ingestion, medications, radiation injury, infection, and malignancy [2,3]. If left untreated, they can cause narrowing of the esophagus and dysphagia [2]. Treatment includes dilation with rubber bougies, wires, or balloons, intralesional steroids, placement of metal self-expanding or plastic stents, or esophagectomy [1-3]. Here, we present a unique case of a 25-year-old uncontrolled diabetic with a recurrent esophageal stricture from longstanding acid reflux that was refractory to treatment.

## Case presentation

**Figure 1:**
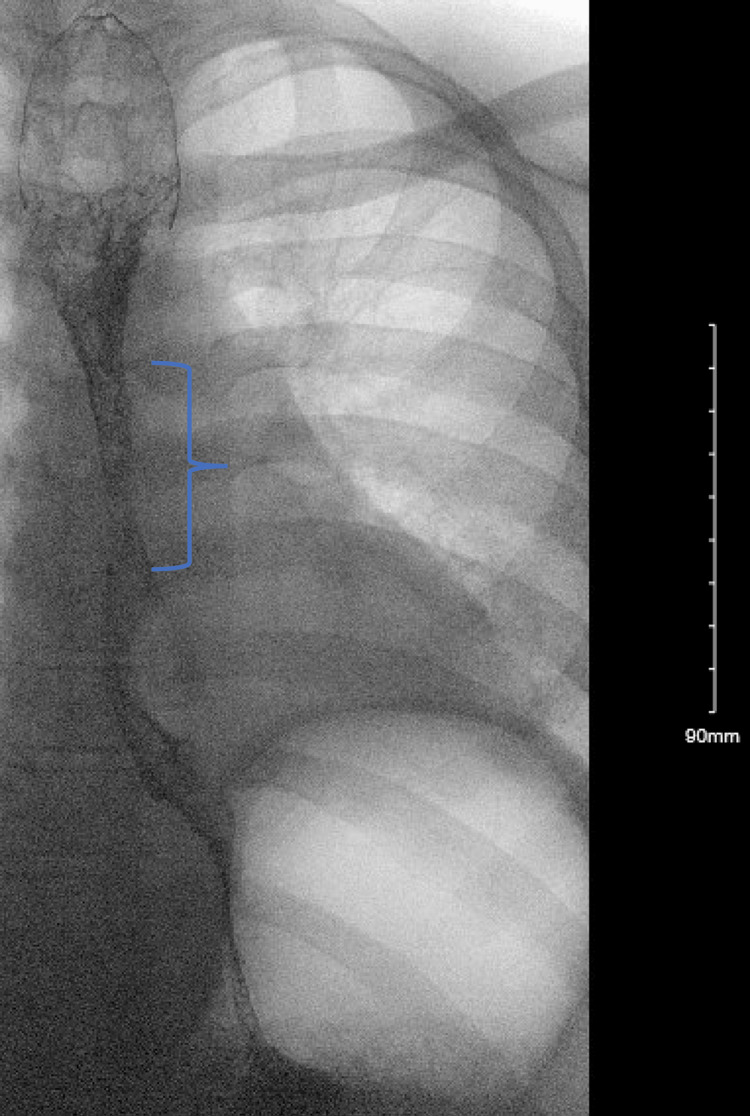
Esophagram showing 5 cm stenosis in the distal esophagus (blue bracket).

Our patient is a 25-year-old male with a history of uncontrolled type 1 diabetes diagnosed 15 years ago who presented with worsening odynophagia and dysphagia. His dysphagia progressed from the inability to swallow solids to liquids. He also had non-bloody, non-bilious emesis several days before presentation. The patient was not taking any medications at the time. His vitals were stable, and the physical exam was unremarkable. His labs were concerning for hemoglobin A1c (HbA1c) of 14.4% (normal 4.8%-5.9%). A barium esophagram showed a 5-centimeter (cm) segment of stenosis at the distal esophagus and signs of reflux without masses or obstruction (Figure 1).

**Figure 2:**
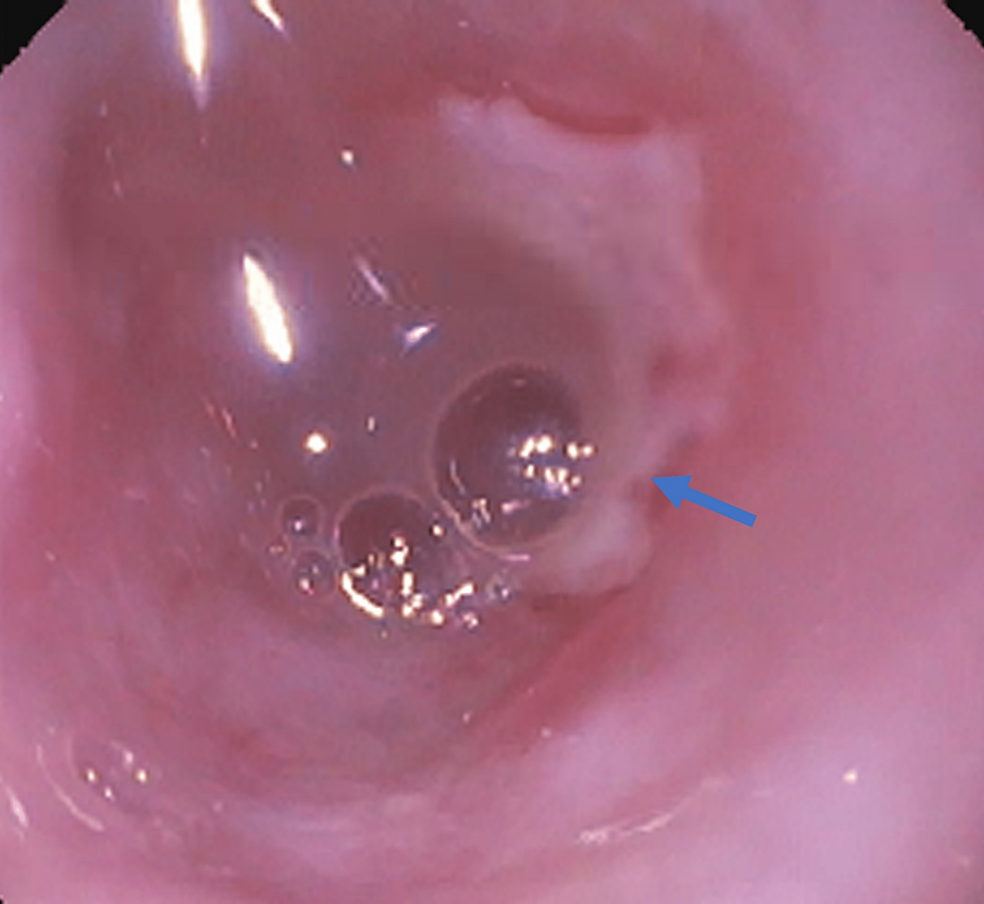
Stricture seen (blue arrow) in the mid-esophagus with near-complete obstruction on EGD.

Esophagogastroduodenoscopy (EGD) was performed using a 9.2-millimeter (mm) outer diameter scope (GIF-H190: Olympus, Shinjuku, Tokyo, Japan) but was unable to traverse the distal esophagus due to near-complete obstruction at 32 cm (Figure 2) from the incisors. An ultrathin endoscope with a 5.4-mm outer diameter (GIF-XP190N: Olympus, Shinjuku, Tokyo, Japan) was able to pass through the 5-cm stenotic segment and the gastroesophageal junction (GEJ) was seen at 37 cm. The distal esophageal lumen was found to be coated with white mucosa that was easily sloughed off. A biopsy of the stenotic area showed sloughing esophagitis but was negative for fungal elements, Herpes Simplex Virus, and Cytomegalovirus. The stomach and the first two portions of the duodenum were normal. The patient was transitioned from intravenous to oral pantoprazole 40 mg daily and discharged home.

**Figure 3:**
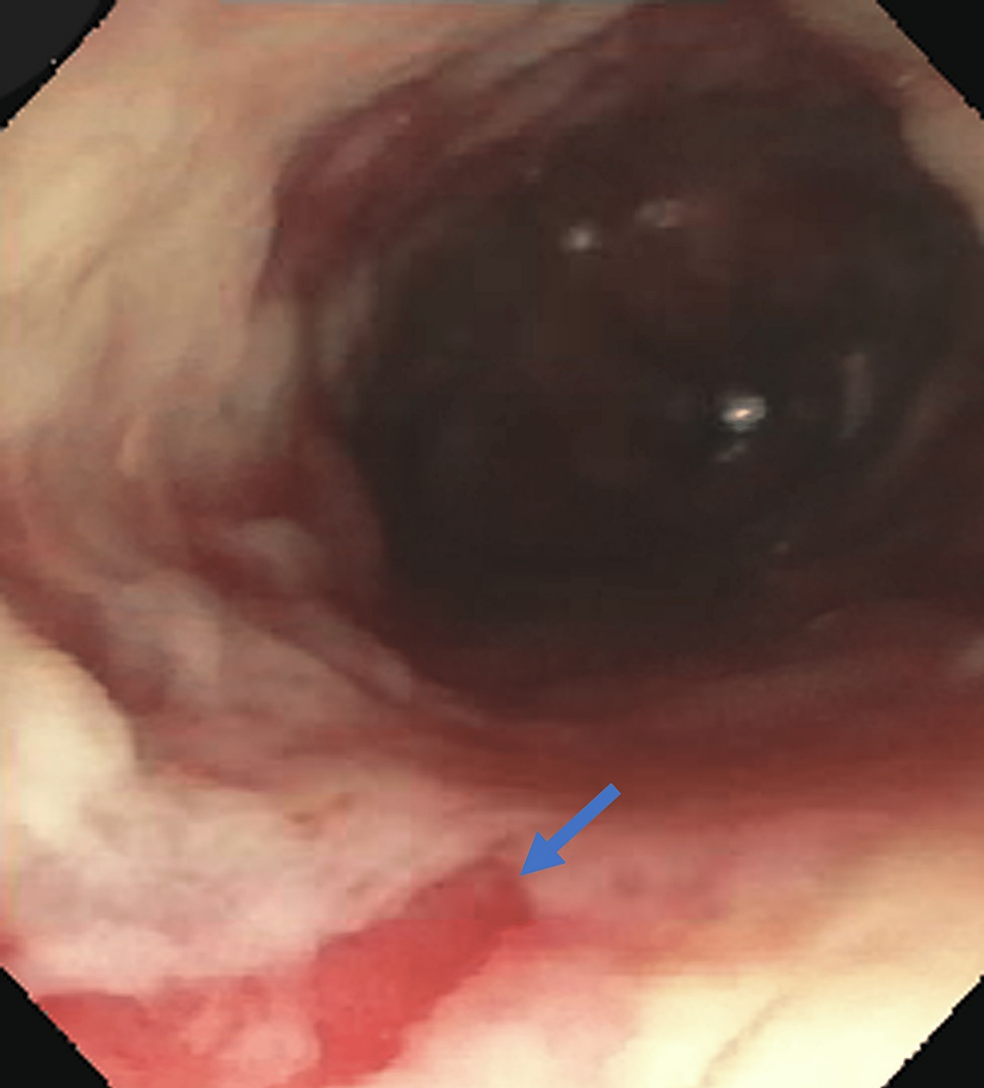
Esophageal lumen with sloughing white mucosa and reflux esophagitis (blue arrow) seen on EGD.

**Figure 4:**
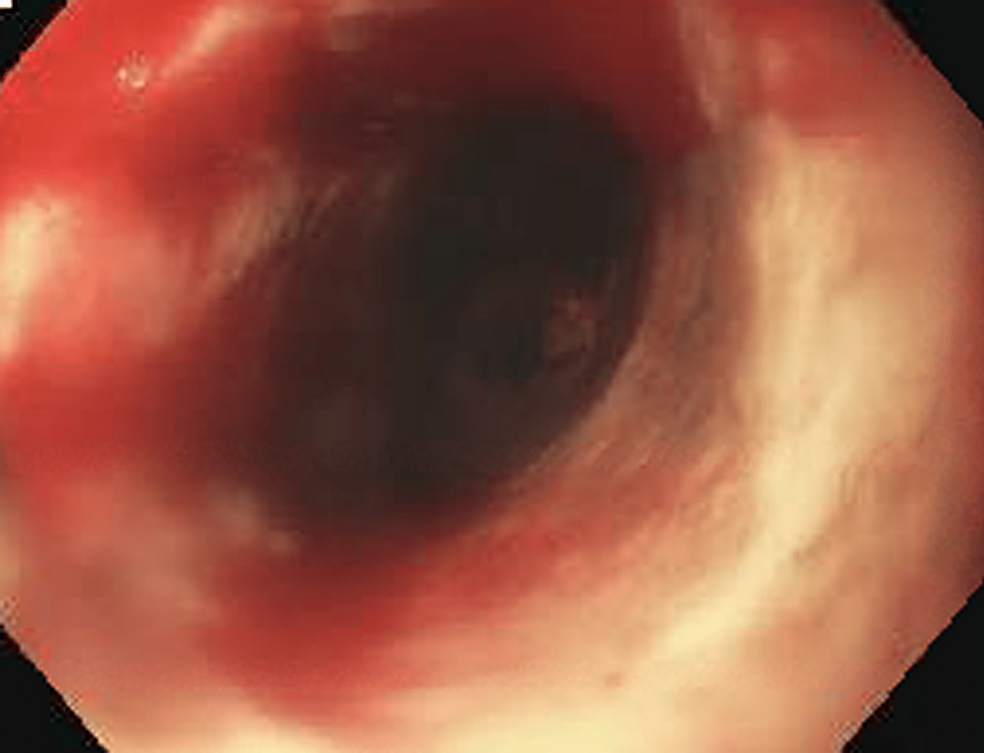
Esophageal lumen after dilation on EGD.

Several weeks later, EGD with balloon dilation was performed using a 12-mm Savary-Gillard guidewire and dilator (Cook Medical, Bloomington IN) with improvement in the patient's symptoms. The colonoscopy was normal and biopsies of the terminal ileum, ascending, transverse, and descending colon were negative for signs of inflammatory bowel disease. However, the patient was admitted to the hospital a month later with recurrent dysphagia to solids and liquids. EGD showed an area of stenosis at the distal esophagus with friable white mucosa from *Candida spp.* seen on pathology (Figures 3, 4) requiring dilation and fluconazole therapy. A stomach biopsy was negative for *Helicobacter pylori*, and a duodenal biopsy showed preserved intraepithelial crypts not suggestive of celiac disease. A gastric emptying study showed 35.6% retention after four hours, consistent with severe delayed gastric emptying so metoclopramide was started. For the next five years, the patient had esophageal dilations every one to two months with Savary-Gillard dilators of up to 18 mm due to symptoms from a recurring esophageal stricture. He required multiple hospitalizations of more than 24 hours for dehydration despite being on maximal therapy of oral pantoprazole and metoclopramide.

The patient was then given a continuous blood glucose monitoring device and an insulin pump to help control his diabetes. His HbA1c decreased to 7.7%, had more persistent improvement in his symptoms, and did not require dilations as frequently. Repeat biopsies sent from the stricture were negative for malignancy or infections. He was ultimately diagnosed with a recurrent esophageal peptic stricture.

## Discussion

Peptic strictures, which are due to acid reflux, account for 70%-80% of esophageal strictures [3-5]. If acid reflux becomes chronic, it can cause repeated esophagitis and eventual scarring of the esophageal mucosa [6]. This can lead to strictures, which are usually shorter than 2 cm and do not extend further than 4 cm from the gastroesophageal junction [6-8]. Strictures can cause stenosis of the esophageal lumen and progressive dysphagia, odynophagia, and weight loss [4,5,9]. Symptoms improve with proton pump inhibitors and dilation, but 50% of patients will need multiple dilations [2,3,7,8]. Dilation is usually done in increments but there is a 0.1%-0.4% risk of perforation with each treatment [1]. Studies have shown that intralesional steroids are beneficial but carry the risk of intramural *Candida albicans* infection, worsening esophagitis, and perforation [1-4]. Stents offer rapid symptomatic relief but are generally reserved for strictures from malignancy and can be displaced, occluded, or lead to fistulas [2,4]. Esophagectomy is the last resort with 5% mortality and 56% risk for postoperative complications [7,8].

Patients with diabetes are 1.25 times more likely to have acid reflux compared to the general population [7]. They can develop peripheral, autonomic, and visceral neuropathy affecting the motility of the gastrointestinal tract manifesting as esophageal dysmotility and gastroparesis [7,10]. Retained stomach contents in gastroparesis cause changes in the thoracoabdominal pressure gradient with subsequent relaxation of the lower esophageal sphincter, leading to reflux [11]. In addition, diabetics are more prone to infections which in itself can exacerbate reflux and esophagitis [3].

**Figure 5:**
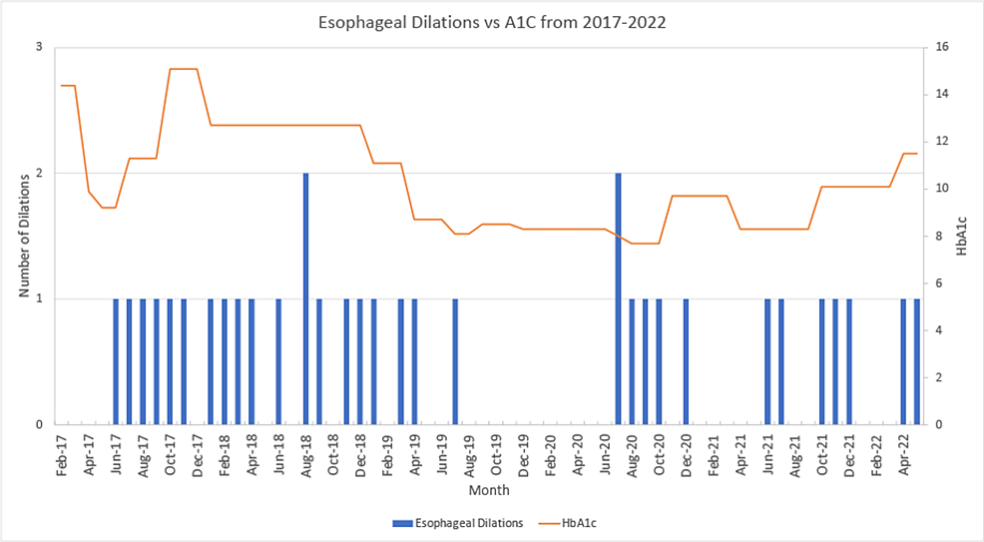
Graph of Esophageal Dilations (blue) vs A1C (orange) for our Patient from 2017 to 2022.

Our patient had longstanding severe diabetes from medication noncompliance. This led him to develop gastroparesis, severe reflux, and an extensive 5-cm stricture with luminal stenosis. There is only one other case report with similar severity of disease described by Pak et al, and Darr et al., who reported a diabetic with a 6-centimeter stricture, failed dilation, and acid suppression therapy requiring esophagectomy [7,8]. However, our case is unique because our patient improved with better control of his diabetes and did not need surgery. The graph (Figure 5) shows the dates our patient had esophageal dilations relative to his HbA1c values from 2017-2022. Using logistic regression analysis in SPSS (IBM Corp. Released 2021. IBM SPSS Statistics for Macintosh, Version 28.0, Armonk, NY), HbA1c is a predictor of esophageal dilations with an odds ratio of 1.42 (95% CI = 1.07-1.87, p=0.014). When his HbA1c was above his average of 10.8%, he received dilations approximately every 36.4 days (SD: 11.1). However, when his HbA1c values were below his average, he needed dilations approximately every 56.7 days (SD: 41.7). This difference was also significant with p = 0.049. The time between 7/2019 to 7/2020 was possibly an anomaly due to the COVID-19 pandemic so it was not included in calculating the number of days between dilations.

Since diabetes can be the inciting factor for peptic strictures, management should include screening for diabetes and focus on adequate blood sugar control in conjunction with acid suppression therapy, gastric motility agents, and endoscopic dilations. This can help minimize symptoms, slow down the formation of esophageal strictures, and decrease the frequency of dilations.

## Conclusions

Peptic strictures in our diabetic patient were difficult to manage because of recurring symptoms and the need for frequent dilations. Diabetes led to gastroparesis, acid reflux, and the formation of a refractory 5 cm esophageal stricture. Further workup for infections, celiac disease, and inflammatory bowel disease was unrevealing, and his symptoms gradually improved with better glycemic control. Besides maximizing therapy with proton pump inhibitors and gastric motility agents, another important aspect of the treatment of peptic strictures is to optimize the management of coexisting diabetes. This can increase the intervals between dilations and therefore improve the quality of life for patients.
